# Relationship between ultrasound damage score of peripheral entheses and spinal bone formation in long-standing radiographic axial spondyloarthritis

**DOI:** 10.1136/rmdopen-2025-006388

**Published:** 2026-03-04

**Authors:** Anna Deminger, Mats Geijer, Magnus Hallström, Lennart T H Jacobsson, Helena Forsblad-d’Elia

**Affiliations:** 1 Department of Rheumatology and Inflammation Research, Institute of Medicine, Sahlgrenska Academy, University of Gothenburg, Gothenburg, Sweden; 2 Department of Rheumatology, Sahlgrenska University Hospital, Gothenburg, Sweden; 3 Department of Radiology, Institute of Clinical Sciences, University of Gothenburg, Gothenburg, Sweden; 4 Department of Radiology, Sahlgrenska University Hospital, Gothenburg, Sweden; 5 Department of Clinical Sciences, Lund University, Lund, Sweden

**Keywords:** Axial Spondyloarthritis, Spondylitis, Ankylosing, Ultrasonography

## Abstract

**Objective:**

To assess structural changes at peripheral entheses and the association with spinal bone formation in patients with long-standing radiographic axial spondyloarthritis (r-axSpA) overall and stratified by sex.

**Methods:**

Peripheral entheses were examined cross-sectionally using ultrasound (US) in patients fulfilling the modified New York criteria for ankylosing spondylitis (AS) and assessed for Outcome Measures in Rheumatology consensus-based structural lesions (enthesophytes, calcifications and erosions) summed to a damage US score (0–42). Spinal radiographs were graded with the modified Stoke AS Spinal Score (mSASSS). Associations between US damage score and mSASSS were assessed with negative binomial regression analyses overall, by sex and by age quartiles.

**Results:**

US was performed in 173 patients, 54% males, with a mean (SD) age of 55 (13) years and symptom duration of 29 (13) years. The prevalence of any structural US lesion was 92%. The US damage score was higher in males than females (mean (SD) 4.7 (3.0) versus 3.3 (2.4), p<0.001) and increased significantly with age, as did mSASSS. Univariate associations between US damage score and mSASSS were found overall and in males, but diminished with older age. The association remained significant for males when the multivariable model was adjusted for symptom duration (rate ratio (95% CI) for log-transformed mSASSS+1: 1.32 (1.04 to 1.67)). Overall, mSASSS was not associated with the US damage score when the multivariable model was adjusted for age or symptom duration.

**Conclusion:**

Structural lesions at the peripheral entheses are common in long-standing r-axSpA and accumulate with age, which may obscure the possible association with spinal bone formation.

**WHAT IS ALREADY KNOWN ON THIS TOPIC**

- Three previous cross-sectional studies on patients with axial spondyloarthritis (axSpA) and one on psoriatic arthritis have shown an independent association between chronic changes at peripheral entheses and spinal bone formation.

**WHAT THIS STUDY ADDS**

- This cross-sectional study, which included older patients compared with most previous studies, demonstrated a univariate association between chronic changes at the peripheral entheses and spinal bone formation. However, this association disappeared when age was taken into account.

**HOW THIS STUDY MIGHT AFFECT RESEARCH, PRACTICE OR POLICY**

- Caution is recommended when using ultrasound chronic changes at the peripheral entheses as a marker of more severe disease regarding spinal bone formation in patients with long-standing radiographic axSpA.

## Introduction

 Ankylosing spondylitis (AS) is a chronic, inflammatory disease that belongs to the family of spondyloarthritis (SpA), a cluster of diseases with common clinical and genetic characteristics. Based on the predominant clinical symptoms, SpA can be grouped into axial SpA (axSpA), with symptoms mainly from the spine and sacroiliac (SI) joints, or peripheral SpA, with symptoms mainly from the peripheral joints and entheses, with a substantial overlap between the groups.^1 2^ AxSpA can be further subdivided based on the presence of structural changes in the SI joints: radiographic axSpA (r-axSpA) if definite sacroiliitis according to the modified New York criteria is observed on radiographs and non-radiographic axSpA (nr-axSpA) when definite sacroiliitis is not detected. Nowadays, the terms r-axSpA and AS are used interchangeably.^3^ AS is characterised by spinal new bone formation with the development of syndesmophytes, in some patients leading to total ankylosis of the spine.^4^ The spinal new bone formation contributes to the impairment of physical function and spinal mobility.^5 6^ Male sex is associated with more spinal bone formation.^7 8^

Enthesitis constitutes inflammation at the skeletal sites where tendons and ligaments are inserted into the bone and is considered a characteristic feature of the SpA family of diseases.^9^ The entheses are subjected to mechanical forces that trigger a local cellular response in the tissues to maintain normal tendon function. If the mechanical loading causes damage to the tissue, an inflammatory response is induced. In the following healing process, ossification at the damaged site can occur^10^ and some entheses are common sites for ectopic bone formation, so-called enthesophytes. Enthesophytes are, however, not specific to SpA.^11^

Ultrasound (US) is a useful, accessible and inexpensive imaging technique for examining the peripheral entheses at the extremities. The Outcome Measures in Rheumatology (OMERACT) US Specialist Interest Group has published a consensus-based definition of the elementary components that constitute US enthesitis in SpA. The definition includes both lesions reflecting active inflammation (Doppler signals, hypoechogenicity and increased thickness) as well as structural, chronic changes (enthesophytes, calcifications and erosions).^12 13^ In recent years, US studies focusing on enthesitis in SpA have attracted a lot of interest. Still, little is known about the relationship between spinal new bone formation and structural changes at the peripheral entheses in axSpA. Four previous studies on this relationship have been published, showing an independent association between structural changes at the entheses and spinal bone formation in AS, axSpA, early inflammatory back pain (IBP) suggestive of SpA and psoriatic arthritis (PsA).^14^,^17^

The objective of this cross-sectional study was to assess the frequencies of structural changes at peripheral entheses and the association between these changes and spinal bone formation in patients with longstanding r-axSpA, overall and stratified by sex.

## Patients and methods

### Patients

The patients were included in 2009 in a longitudinal study with a focus on osteoporosis.^18^ All patients with a diagnosis of AS at three rheumatology clinics in western Sweden were screened for eligibility and invited to participate if they met the study criteria. Inclusion criteria were AS according to the modified New York criteria^19^ and age≥18 years. Exclusion criteria were psoriasis, inflammatory bowel disease (IBD), dementia, ongoing pregnancy and difficulties in understanding the Swedish language. Data for this present cross-sectional study were collected at the 5-year follow-up visit in 2014. The inclusion process for the 173 patients who underwent ultrasonography, and of whom 169 underwent radiography, has been described in detail previously.^20^

The patients answered questionnaires about sex, medical history, smoking, present occupation and medications. Leisure-time physical activities in hours per week during the last month were estimated using a modification of the Leisure Time Physical Activity Instrument (LTPAI).^21^ The modification summed moderate activities (affecting breathing and/or sweating to some extent) and heavy activities (significantly affecting both), excluding activities with no impact on breathing. Occupation was categorised in blue-collar work, involving manual labour and physical tasks, and white-collar work, usually more sedentary and office-based work.^22^ Patients were also assessed with the Bath AS Disease Activity Index (BASDAI), the AS Disease Activity Score (ASDAS) based on C reactive protein (CRP) and the Bath AS Functional Index. Back and hip mobility was evaluated with Bath AS Metrology Index.^23^

Height and weight were measured to calculate body mass index (BMI).

High-sensitivity CRP was analysed using standard laboratory techniques.

### Ultrasonography

**Figure 1:**
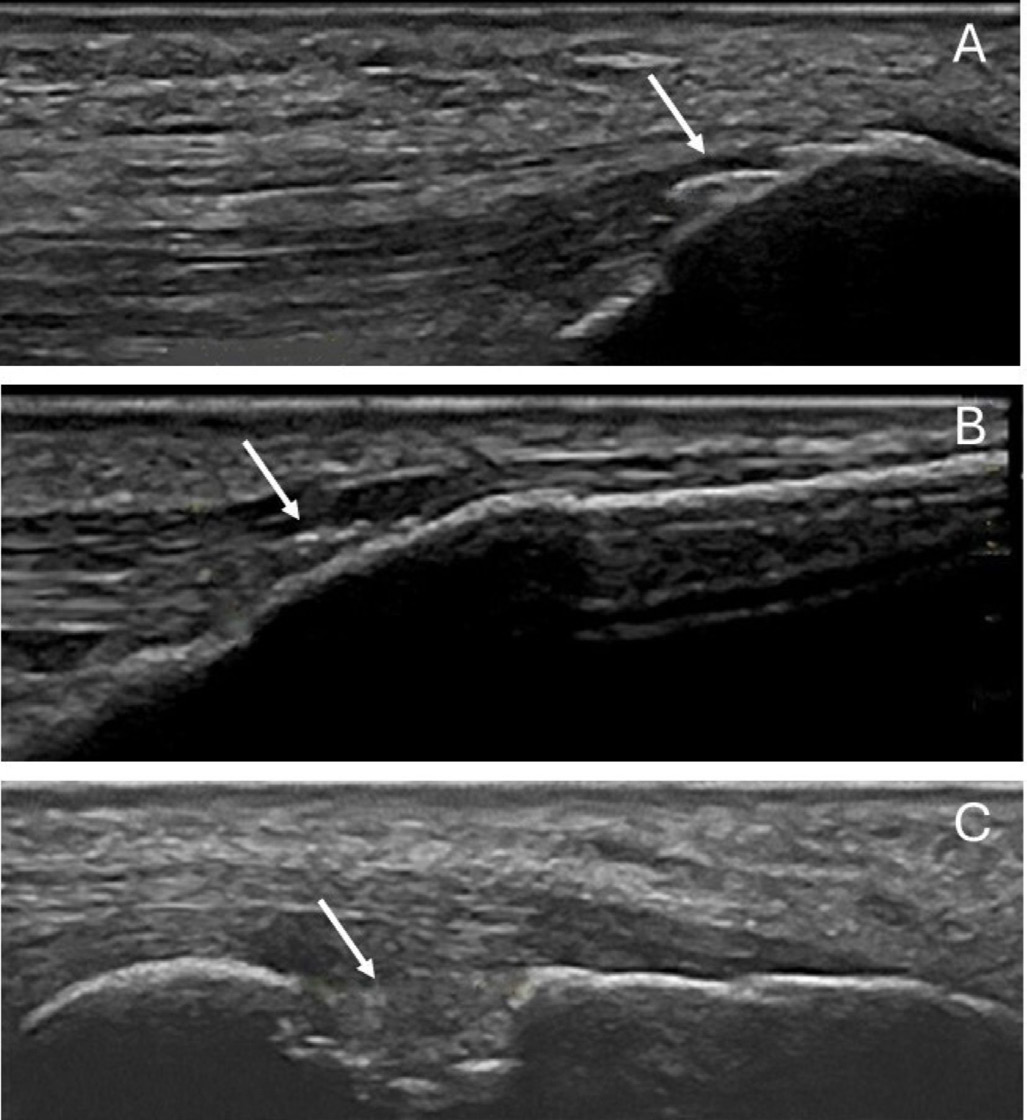
Ultrasound images of (A) enthesophytes at the quadriceps tendon insertion on the patella. (B) Calcifications at the patellar ligament insertion on the tibia. (C) Erosion at the Achilles tendon insertion on the calcaneus.

One trained rheumatologist (AD) performed musculoskeletal ultrasonography using a Logic P6 (General Electric, Boston, Massachusetts, USA) machine equipped with a 6–15 MHz linear transducer. The sonographer was blinded to radiological but not to clinical results. The following entheses were examined bilaterally: the lateral humeral epicondyle, the triceps tendon insertion on the olecranon process, the quadriceps tendon insertion on the patella, the patellar tendon insertion on the patella and the tibial tuberosity, the Achilles tendon and the plantar fascia insertion on the calcaneus. In a supine position with the elbow flexed to 90° and the knee flexed to 30°, the patient was examined at the entheses of the lateral humeral epicondyle, the triceps, the quadriceps and the patella. The examination at the calcaneus was undertaken with the patient in a prone position, with the feet hanging in a neutral position over the edge of the examination table. Adjustments to the US machine settings were performed as needed to achieve optimal image acquisition. Each enthesis was evaluated immediately for the presence of the structural components enthesophyte, calcification and erosion according to the consensus-based definitions by OMERACT (figure 1).^12^ Each type of structural component was defined as present/absent at each entheseal site and summed up to a total US damage score, 0–42. Images were stored for each entheseal site for all patients. If data were missing for an entheseal site, that site was counted as 0 in the calculation of the US damage score.

### Radiography

At a separate visit, lateral radiographs of the cervical and lumbar spine were obtained for the grading of AS-related spinal radiographic alterations according to the modified Stoke Ankylosing Spondylitis Spinal Score (mSASSS). The score ranges from 0 to 72.^24^ The grading was performed by one musculoskeletal radiologist (MG).

### Statistical analyses

Descriptive statistics are presented according to their distribution; normally distributed variables are presented as means with SD, skewed variables as both medians with 25th–75th percentile and means (SD), and frequencies as numbers with percentages. Variables were compared between sexes with the t*-*test, the Mann-Whitney U-test, the χ^2^ test or Fisher’s exact test, as appropriate. Physical activity+1 and mSASSS+1 were log-transformed to normalise the distributions. The dependent variable, US damage score, representing the sum of the number of lesions at the entheses, was modelled using negative binomial regression analyses to account for overdispersion (the variance exceeded the mean of the total score). First, univariate analyses were performed for the total group and by sex. Independent variables were selected based on existing knowledge of factors associated with chronic changes at the entheses and spinal bone formation. Then, variables were considered for the multivariable analyses if the p value in the univariate analysis was ≤0.1. Collinearity was assessed for the multivariable regression analyses. Correlations between the independent variables were analysed using Pearson’s correlation coefficient. If the correlation was high (>0.6 or <−0.6), the variables were not used in the same model. The models were evaluated with a goodness-of-fit test. Missing data were handled with listwise deletion. In the overall model, potential interactions between mSASSS and age and between mSASSS and sex were assessed, but none reached statistical significance. Sensitivity analyses excluding the plantar fascia from the US damage score were conducted. The correlations between the US damage score and mSASSS were analysed using Spearman’s correlation coefficient (R_S_) in patients stratified by age quartiles. Age quartiles 2 and 3 had similar R_S_ and were combined for subsequent analyses with univariate and multivariable negative binomial regression models. The Kruskal-Wallis test was used to compare mSASSS and US damage scores across age quartiles.

Tests were two-tailed, and p≤0.05 was considered statistically significant. All statistical analyses were conducted using IBM SPSS Statistics V.28, except for the goodness-of-fit tests for the regression analyses, which were performed using SAS for Windows V.9.4.

#### Reliability

Intrareader agreement for grading of spinal bone formation with mSASSS has been published previously with an intraclass correlation coefficient (ICC) for a status score of 0.98 (95% CI 0.96 to 0.99).^20^

The intrareader agreement for the US damage score was assessed on stored images of 40 randomly selected patients. In total, 530 images were evaluated twice, 4 months apart, for calculation of the ICC two-way mixed-effect model, single measurement and absolute agreement. Values <0.50 indicate poor agreement, between 0.50 and 0.75 moderate agreement, between 0.75 and 0.90 good agreement and >0.90 excellent agreement.^25^

## Results

### Patients

In total, 173 patients (94 males and 79 females) were included in this study and underwent ultrasonography of the entheses, whereas 169 patients underwent radiography. The mean (SD) interval between the ultrasonography and the radiography was 200 (75) days. The mean (SD) age was 55 (13) years, and the mean symptom duration was 29 (13) years, with no differences between males and females. The characteristics and medications overall and with comparisons between sexes are shown in table 1. Males had higher BMI, and more males were human leucocyte antigen B27 (HLA-B27) positive compared with females. Furthermore, a higher proportion of males than females had the presence of at least one syndesmophyte, 64% versus 36% (p<0.001) of the radiographed patients and males exhibited more spinal radiographic alterations according to mSASSS, with a mean (SD) of 23.8 (23.8) versus 7.6 (11.7) in females (p<0.001).

**Table 1:** Characteristics and medication in 173 patients with radiographic axial spondyloarthritis overall and with a comparison between sexes

	Total group	Males (n=94)	Females (n=79)	P value
Demographic variables				
Age, years	55 (13)	54 (13)	56 (13)	0.37
BMI, kg/m^2^	26.0 (23.3–29.6)	27.3 (24.6–30.0)	25.0 (22.6–28.7)	**0.004**
	27.0 (5.0)	27.9 (4.9)	26.0 (4.9)	
Current or past smoking	83 (48)	46 (49)	37 (47)	0.90
Present blue-collar worker, yes	32 (28)*	21 (31)^†^	11 (23)^‡^	0.41
Physical activity, hours/week	3.5 (2–6)	4 (2–6)	3 (1.5–5.5)	0.51
	4.3 (3.9)	4.5 (4.1)	4.1 (3.7)	
Disease-related variables				
Symptom duration, years	29 (13)^§^	28 (13)^¶^	30 (13)	0.42
HLA-B27 positive, yes	150 (87)	87 (93)	63 (80)	**0.025**
BASMI, score	3.5 (1.6)	3.6 (1.8)	3.4 (1.3)	0.45
BASFI, score	2.3 (1.0–4.2)	2.3 (1.0–4.0)	2.4 (1.2–4.5)	0.40
	2.8 (2.2)	2.7 (2.1)	2.9 (2.2)	
BASDAI, score	3.5 (2.0)^**^	3.0 (1.9)	4.2 (2.0)^††^	**<0.001**
ASDAS, score	2.1 (0.9)^**^	2.0 (0.9)	2.2 (0.9)^††^	0.068
CRP, mg/L	3 (1–6)	3 (1–7)	2 (1–5)	0.75
	4.6 (5.3)	4.8 (5.2)	4.4 (5.3)	
mSASSS, (0–72)	7 (0–23.5)^‡‡^	14 (3–43)^¶^	2 (0–11.5)^§§^	**<0.001**
	16.4 (20.8)^‡‡^	23.8 (23.8)**^¶^**	7.6 (11.7)^§§^	
≥1 syndesmophyte, yes	87 (51)^‡‡^	59 (64)^¶^	28 (36)^§§^	**<0.001**
Use of NSAIDs, yes	115 (66.5)	61 (65)	54 (68)	0.75
Use of TNFi, yes	38 (22)	26 (28)	12 (15)	0.074

Values are presented as mean (SD), median (25th–75th percentile) or number (%). Skewed variables are presented with both median (25th–75th percentile) and mean (SD).

Comparisons between sexes with the t-test, the Mann-Whitney U-test, the χ2 test or Fisher’s exact test as appropriate.

P values ≤0.05 are highlighted in bold.

* n=115.

† n=68.

‡ n=47.

§ n=171.

¶ n=92.

** n=172.

†† n=78.

‡‡ n=169.

§§ n=77.

ASDAS, Ankylosing Spondylitis Disease Activity Score; BASDAI, Bath Ankylosing Spondylitis Disease Activity Index; BASFI, Bath Ankylosing Spondylitis Functional Index; BASMI, Bath Ankylosing Spondylitis Metrology Index; BMI, body mass index; CRP, C reactive protein; HLA-B27, human leucocyte antigen B27; mSASSS, modified Stoke Ankylosing Spondylitis Spinal Score; NSAID, non-steroidal anti-inflammatory drug; TNFi, tumour necrosis factor inhibitor.

### Presence of US chronic lesions and US damage score

A total of 159 patients (92%) had one or more structural lesions at the extremities, with no significant difference between males and females, 95% versus 89%, respectively (table 2). In the total group, structural lesions occurred more often in the lower extremities compared with the upper extremities; 91% of the patients had any structural lesion at the entheses in the legs versus 45% in the arms, p=0.007. A significant difference between males and females in the occurrence of structural lesions was found at the upper extremities; 52% of males had any lesion at the arm compared with 37% of females (table 2).

**Table 2:** Presence of any structural lesion at the extremities, frequencies of type of lesions and total ultrasound damage score overall, and with a comparison between sexes

	Total group (n=173)	Males (n=94)	Females (n=79)	P value
Structural lesion				
Any structural lesion arm	78 (45.1)	49 (52.1)	29 (36.7)	**0.042**
Any structural lesion leg	157 (90.8)	88 (93.6)	69 (87.3)	0.19
Any structural lesion total	159 (91.9)	89 (94.7)	70 (88.6)	0.17
Enthesophyte	159 (91.9)	89 (94.7)	70 (88.6)	0.17
Calcification	68 (39.3)	41 (43.6)	27 (34.2)	0.27
Erosion	12 (6.9)	8 (8.5)	4 (5.1)	0.55
Total US damage score	4.1 (2.8)	4.7 (3.0)	3.3 (2.4)	**<0.001**

Values are number of patients (%) or mean (SD). Comparison between sexes with the χ2 test, Fisher’s exact test or the t*-*test.

P values ≤0.05 are highlighted in bold.

US, ultrasound.

The most common lesion observed was an enthesophyte, occurring in 92% of the patients, whereas calcifications and erosions were found in 39% and 7%, respectively (table 2). The most common entheseal site for structural lesions was the insertion of the quadriceps tendon into the patella, with 86.5% of the total group having an enthesophyte at that site. The second and third most common sites for chronic lesions were the insertion of the Achilles tendon on the calcaneus and the insertion of the triceps tendon, with 54% and 34% of the patients with an enthesophyte at those sites, respectively (table 3). Enthesophytes were more common in males compared with females at all entheseal sites except for the lateral humeral epicondyle, the proximal patella and the plantar fascia (table 3).

**Table 3:** Presence of ultrasound structural lesions in patients with radiographic axial spondyloarthritis at seven separate entheseal sites overall and by sex

Entheseal site	Lesion	Total (n=173)	Males (n=94)	Females (n=79)	P value
Lateral humeral epicondyle	Enthesophyte	19 (11.0)	9 (9.6)	10 (12.7)	0.63
	Calcification	4 (2.3)	2 (2.1)	2 (2.5)	NP
	Erosion	0 (0)	0 (0)	0 (0)	NP
Triceps tendon	Enthesophyte	59 (34.1)	41 (43.6)	18 (22.8)	**0.007**
	Calcification	14 (8.1)	11 (11.7)	3 (3.8)	NP
	Erosion	1 (0.6)	0 (0)	1 (1.3)	NP
Quadriceps tendon*	Enthesophyte	148 (86.5)	86 (92.4)	62 (79.4)	**0.024**
	Calcification	45 (26.3)	26 (28.0)	19 (24.4)	NP
	Erosion	0 (0)	0 (0)	0 (0)	NP
Proximal patellar tendon†	Enthesophyte	34 (19.8)	22 (23.7)	12 (15.2)	0.23
	Calcification	2 (1.2)	2 (2.2)	0 (0)	NP
	Erosion	0 (0)	0 (0)	0 (0)	NP
Distal patellar tendon	Enthesophyte	25 (14.5)	19 (20.2)	6 (7.6)	**0.033**
	Calcification	9 (5.2)	5 (5.3)	4 (5.1)	NP
	Erosion	0 (0)	0 (0)	0 (0)	NP
Achilles tendon	Enthesophyte	93 (53.8)	59 (62.8)	34 (43.0)	**0.015**
	Calcification	11 (6.4)	10 (10.6)	1 (1.3)	NP
	Erosion	7 (4.0)	6 (6.4)	1 (1.3)	NP
Plantar fascia‡	Enthesophyte	18 (11.3)	8 (10.0)	10 (12.7)	0.78
	Calcification	3 (1.9)	1 (1.3)	2 (2.5)	NP
	Erosion	4 (2.5)	2 (2.5)	2 (2.5)	NP

Values are number of patients (%). Comparison of the presence of enthesophytes between sexes with the χ2 test or Fisher’s exact test.

P values ≤0.05 are highlighted in bold.

* Quadriceps total n=171, men n=93, women n=78.

† Proximal patella total n=172, men n=93, women n=79.

‡ Plantar fascia total n=159, men n=80, women n=79.

NP, no statistical tests performed.

The total US damage score ranged from 0 to 14 in the total group. The mean (SD) score was 4.1 (2.8) with a significantly higher score in males compared with females, 4.7 (3.0) versus 3.3 (2.4), respectively (table 2).

#### Reliability of the US damage score

The ICC (95% CI) for the analysis of the intrareader agreement of the US damage score on the stored images was 0.90 (0.82 to 0.95).

### Factors associated with US damage score

#### Univariate negative binomial regression analyses

Factors associated with US damage score were studied in the 169 patients with spinal radiographs, 92 males and 77 females (table 4). In the total group, univariate regression analyses revealed that a higher US damage score was significantly associated with being male, older age, a higher BMI, longer symptom duration and more spinal bone formation (assessed by mSASSS). There were no significant associations between US damage score and smoking status, physical activity, HLA-B27, use of TNFi, disease activity or CRP. Within the subgroup of patients who remained in employment, no significant association was observed for blue-collar work and US damage score.

**Table 4:** Univariate negative binomial regression analyses assessing factors associated with ultrasound damage score overall and by sex in patients with radiographic axial spondyloarthritis and spinal radiographs

	Total group, n=169		Males, n=92		Females, n=77	
	RR (95% CI)	P value	RR (95% CI)	P value	RR (95% CI)	P value
Sex, males	1.44 (1.17 to 1.78)	**<0.001**	NA		NA	
Age, years	1.02 (1.02 to 1.03)	**<0.001**	1.02 (1.01 to 1.03)	**<0.001**	1.03 (1.02 to 1.04)	**<0.001**
BMI, kg/m^2^	1.02 (1.00 to 1.04)	**0.028**	1.01 (0.98 to 1.04)	0.54	1.03 (1.00 to 1.06)	**0.048**
Smoking, yes	1.06 (0.86 to 1.31)	0.58	1.19 (0.92 to 1.55)	0.18	0.85 (0.61 to 1.18)	0.33
Present blue-collar work, yes	0.89 (0.65 to 1.20)*	0.44	NP		NP	
Log (physical activity+1), hours/week	0.78 (0.56 to 1.10)	0.15	0.70 (0.46 to 1.06)	0.091	0.86 (0.51 to 1.45)	0.58
Symptom duration, years	1.01 (1.01 to 1.02)	**0.001**	1.02 (1.01 to 1.03)	**0.005**	1.01 (1.00 to 1.03)	**0.031**
HLA-B27, positive, yes	0.89 (0.66 to 1.21)	0.47	0.76 (0.48 to 1.21)	0.24	0.83 (0.56 to 1.23)	0.36
Use of TNFi, yes	0.98 (0.76 to 1.26)	0.85	0.94 (0.71 to 1.27)	0.70	0.83 (0.52 to 1.33)	0.45
BASDAI, score	1.01 (0.96 to 1.06)	0.78	1.07 (1.00 to 1.14)	0.066	0.96 (0.92 to 1.09)	0.96
CRP, mg/L	1.00 (0.98 to 1.02)	0.87	0.99 (0.97 to 1.02)	0.56	1.01 (0.98 to 1.04)	0.41
Log (mSASSS+1), score	1.39 (1.19 to 1.62)	**<0.001**	1.31 (1.06 to 1.61)	**0.012**	1.29 (0.98 to 1.69)	0.073

RR is the rate ratio (Exp (β)), where β models the natural logarithm of the expected count in the ultrasound damage score.

P values ≤0.05 are highlighted in bold.

* n=112.

BASDAI, Bath Ankylosing Spondylitis Disease Activity Index; BMI, body mass index; CRP, C reactive protein; HLA-B27, human leucocyte antigen B27; Log, log-transformed; mSASSS, modified Stoke Ankylosing Spondylitis Spinal Score; NA, not applicable; NP, sex-stratified analyses not performed due to low statistical power; TNFi, tumour necrosis factor inhibitor.

In males, the same variables were significantly associated with the US damage score as in the total group, except for BMI, which showed no significant association. In females, older age, longer symptom duration and higher BMI were significantly associated with higher US damage score, whereas spinal bone formation did not reach significance, although the rate ratio (RR) was very similar to that observed in males.

#### Multivariable negative binomial regression analyses

In the multivariable regression analysis for the total group, a higher US damage score was independently associated with older age and male sex, and no longer significantly associated with BMI or spinal bone formation (table 5, model 1).

**Table 5:** Multivariable negative binomial regression analyses assessing factors associated with ultrasound damage score overall and by sex in patients with radiographic axial spondyloarthritis and spinal radiographs

	Total group, n=169		Males, n=92		Females, n=77	
	RR (95% CI)	P value	RR (95% CI)	P value	RR (95% CI)	P value
Model 1						
Intercept	0.68 (0.36 to 1.29)	0.24	1.66 (0.88 to 3.16)	0.12	0.36 (0.14 to 0.91)	**0.030**
Sex, male	1.47 (1.19 to 1.81)	**<0.001**	NA		NA	
Age, years	1.02 (1.02 to 1.03)	**<0.001**	1.02 (1.01 to 1.03)	**0.003**	1.03 (1.02 to 1.04)	**<0.001**
BMI, kg/m^2^	1.01 (0.99 to 1.03)	0.41	NM		1.02 (1.00 to 1.05)	0.10
Log (physical activity+1), hours/week	NM		0.81 (0.56 to 1.19)	0.29	NM	
BASDAI, score	NM		1.04 (0.97 to 1.11)	0.29	NM	
Log (mSASSS+1), score	1.01 (0.85 to 1.21)	0.89	1.07 (0.85 to 1.35)	0.56	1.02 (0.80 to 1.29)	0.90
	Total group, n=167		Males, n=90		Females, n=77	
	RR (95% CI)		RR (95% CI)		RR (95% CI)	
Model 2						
Intercept	1.49 (0.83 to 2.68)	0.19	2.88 (1.80 to 4.60)	**<0.001**	0.90 (0.36 to 2.23)	0.82
Sex, male	1.28 (1.02 to 1.60)	**0.033**	NA		NA	
Symptom duration, years	1.01 (1.00 to 1.02)	**0.014**	1.01 (1.00 to 1.02)	0.24	1.01 (1.00 to 1.03)	**0.047**
BMI, kg/m^2^	1.01 (1.00 to 1.03)	0.16	NM		1.03 (1.00 to 1.06)	**0.031**
Log (physical activity+1), hours/week	NM		0.76 (0.51 to 1.11)	0.16	NM	
BASDAI, score	NM		1.05 (0.98 to 1.12)	0.17	NM	
Log (mSASSS+1), score	1.18 (0.97 to 1.44)	0.090	1.32 (1.04 to 1.67)	**0.024**	1.07 (0.80 to 1.45)	0.64

RR is the rate ratio (Exp (β)), where β models the natural logarithm of the expected count in the ultrasound damage score.

P values ≤0.05 are highlighted in bold.

Model 1 is adjusted for age, and Model 2 for symptom duration; otherwise, the same independent variables are used.

BASDAI, Bath Ankylosing Spondylitis Disease Activity Index; BMI, body mass index; Log, log-transformed; mSASSS, modified Stoke Ankylosing Spondylitis Spinal Score; NA, not applicable; NM, not used in the model due to p value>0.1 in the univariate analysis.

Age and symptom duration were highly correlated (Pearson’s correlation coefficient=0.72) and were not used in the same model. If age was replaced by symptom duration, mSASSS was still not significantly associated with the US damage score (table 5, model 2).

In males, older age was independently associated with a higher US damage score in a model that included age, physical activity, BASDAI and spinal bone formation (table 5, model 1). In the model with symptom duration, log-transformed mSASSS+1 was significantly associated with the US damage score, RR (95% CI) 1.32 (1.04 to 1.67), p=0.024 (table 5, model 2).

In females, older age, longer symptom duration and higher BMI were independently associated with a higher US damage score (table 5, models 1 and 2).

#### Further analyses regarding age and US damage score

If age was excluded from the multivariable regression model for the total group, spinal bone formation became significantly associated with the US damage score. For log-transformed mSASSS+1, RR (95% CI) was 1.27 (1.07 to 1.51), p=0.007 (online supplemental table 1).

**Figure 2:**
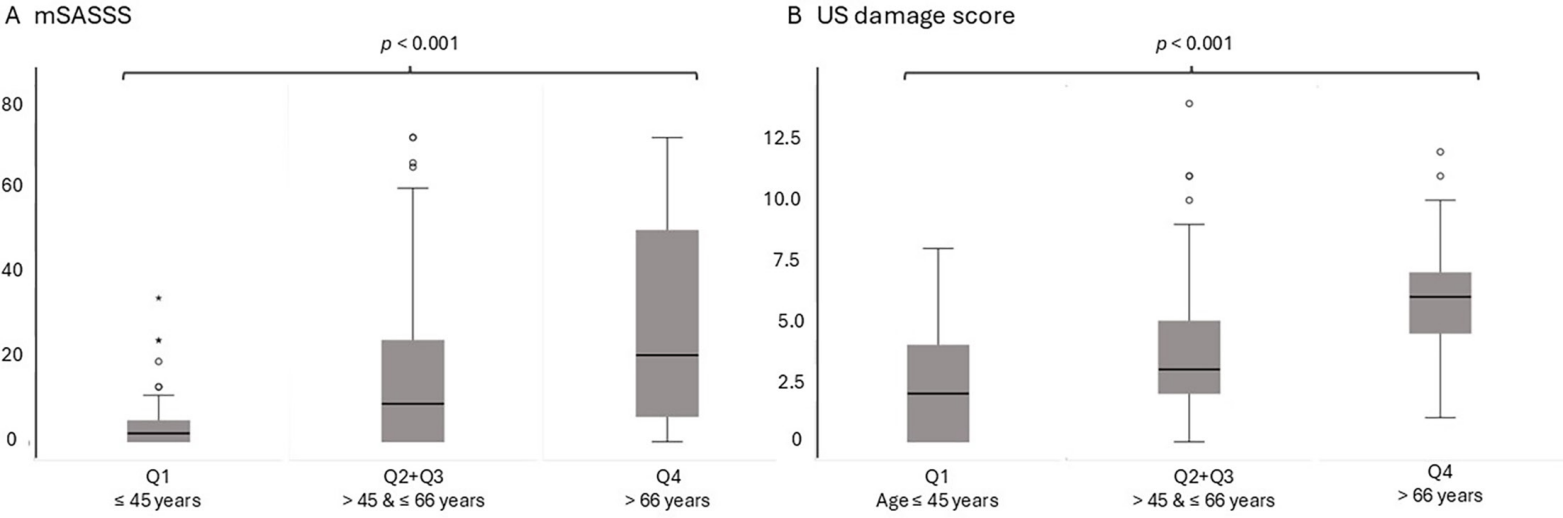
Boxplots demonstrating increasing (A) mSASSS and (B) ultrasound damage score across age quartiles for Q1, Q2+Q3 and Q4. mSASSS, modified Stoke Ankylosing Spondylitis Spinal Score; Q, quartile; US, ultrasound.

When the patients were stratified by quartiles based on age (Q1–4), no correlations between US damage score and mSASSS were detected in the youngest and oldest quartiles (Q1 and Q4), with the Spearman’s correlation coefficients (R_S_) of 0.057 (p=0.71) and 0.012 (p=0.94), respectively. For Q2 and Q3, the R_S_ were similar; R_S_ for Q2 was 0.23 (p=0.13) and for Q3 0.28 (p=0.089), and these two groups were combined for further analyses (Q2+Q3). The characteristics of Q1, Q2+Q3 and Q4 are shown in online supplemental table 2. For Q1, Q2+Q3 and Q4, the median mSASSS increased from 2.0 to 9.0 and 20.5, whereas the corresponding US damage scores increased from 2.0 to 3.0 and 6.0 (figure 2).

Univariate negative binomial regression analyses regarding factors associated with US damage score for Q1, Q2+Q3 and Q4 are presented in online supplemental table 3. For Q2+Q3, there was a significant association between the US damage score and log-transformed mSASSS+1, RR (95% CI) 1.35 (1.10 to 1.64), p=0.004. This association was not significant in Q1 or Q4 (RR (95% CI) 1.11 (0.56 to 2.20) and 1.02 (0.82 to 1.25), respectively). In a multivariable model for Q2+Q3 that included sex, BMI and log-transformed mSASSS+1, male sex was associated with a higher US damage score, whereas spinal bone formation was not significantly so (RR (95% CI) 1.18 (0.95 to 1.47), p=0.15) (online supplemental table 4).

#### Multivariable sensitivity analyses

When sensitivity analyses were conducted using the US damage score excluding the plantar fascia, significant associations were found between the score and age and male sex in the total group, as well as with age in the male subgroup (online supplemental table 5).

## Discussion

This cross-sectional US study of 173 patients with long-standing r-axSpA found a high prevalence of structural lesions at the entheses. The total US damage score was higher in males than in females and was independently associated with the male sex. There were significant associations between US-determined structural changes at the peripheral entheses and spinal bone formation in univariate analyses, overall and in males. In multivariable models, the association remained significant for males when the model was adjusted for symptom duration, whereas the association did not persist overall when adjusting for age or symptom duration.

Three previous cross-sectional studies on axSpA^14 16 17^ and one on PsA (patients underwent X-rays of the SI joints, and 37% had sacroiliitis, but axial symptoms were not reported^15^) have shown an independent association between structural changes at the entheses and spinal bone formation. All studies, including our present study, have had some differences in the scoring method, and there is currently no consensus on which US scoring method to use in SpA, on how to grade abnormalities or on which entheses to include. Also, the multivariable models differed in the outcome variable and variables included for adjustment. Aydin *et al* studied patients with AS and focused on enthesophytes at the insertion of the Achilles tendon using a semiquantitative US enthesophyte score, with the outcome variable presence of ≥1 syndesmophyte in the spine among 200 patients. They found an independent association for the enthesophyte score in the overall group and specifically in males, in a model adjusted for age, disease duration and BMI.^14^ Solmaz *et al* evaluated the same structural components at the same entheseal sites as we did, but they used a semiquantitative US damage score in their study population of patients with AS, nr-axSpA, axial PsA and SpA related to IBD. This is the only previous study that has used US damage score as the dependent variable in one of the analyses. They found an independent association with mSASSS among 71 patients in a model adjusted for age, sex, BMI, disease duration, psoriasis, arthritis, dactylitis, current treatment with biologic disease-modifying antirheumatic drugs (bDMARDs) and HLA-B27.^17^ Patients with PsA had the strongest correlation between mSASSS and US damage score, and psoriasis was independently associated with US damage score, as well as older age, male sex and higher BMI. Further, Ruyssen-Witrand *et al* included patients with early IBP suggestive of axSpA.^16^ Similar to our study, they recorded the presence of abnormalities and summed them up. However, they included not only enthesophytes, calcifications and erosions in their structural score but also abnormal thickness and Achilles’ bursitis. They assessed four entheseal sites bilaterally. Their outcome was mSASSS≥2 in 358 patients, and they reported an independent association for the US structural score in a model that also included age, sex, BMI, HLA-B27, ASDAS-CRP, sacroiliitis on MRI, smoking status, disease duration and US power Doppler score. The association with spinal bone formation was stronger, although a large CI indicates less precision, when the presence of ≥1 enthesophyte was analysed instead of the US structural score.^16^ Last, Polachek *et al* performed US on the same entheseal sites as we did, except not including the lateral epicondyle, in 233 patients with PsA. They found an independent association between the semiquantitative bone score (erosions and calcifications/enthesophytes) and the outcome mSASSS in a model adjusted for age, sex, disease duration, BMI, smoking, current use of DMARDs and bDMARDs.^15^ In the present study, we chose the most feasible scoring method of recording the presence of abnormalities. This method is applicable in clinical practice, whereas semiquantitative scoring is more difficult to apply, especially since there is no endorsed scoring method, and no atlas has been published. Independent variables in the present study were selected based on existing knowledge of factors related to structural entheseal changes, and since such data are limited, factors associated with spinal bone formation were also used.

Besides methodological differences between prior studies on US damage and the relationship with spinal bone formation and our study, there are some other differences worth noting. Our patients with long-standing r-axSpA had more spinal radiographic alterations than the reported mean or median mSASSS in all previously mentioned studies. Due to differences in methodologies, it is difficult to compare the prevalences and extent of structural entheseal alterations between studies.^14^,^17^ Additionally, our patients with a mean age of 55 years were older than the participants in the previous studies, except for the study on PsA by Polachek *et al* with a mean (SD) age of 56 (13) years.^15^ The median age reported by Ruyssen-Witrand and Aydin was 33 and 34 years, respectively, and the mean (SD) age reported by Solmaz was 45 (13) years.^14 16 17^ Furthermore, older age was independently associated with higher US damage scores in our present study. This independent association has previously been reported for axSpA and PsA.^17 26^ Likewise, studies on healthy controls have shown associations between older age and higher US damage scores.^27 28^ The regression slopes for the US scores differed between participants younger and older than 50 years in the study by Bakirci *et al*, with an increased slope in the older participants.^27^ In the present study, spinal bone formation was univariately associated with the US damage score, and when age was not included in the multivariable model. Further, the association between spinal bone formation and chronic changes at the entheses differed across age quartiles. The lack of association in the youngest quartile could be attributed to a relatively low occurrence of abnormalities, whereas the absence of association in the oldest quartile is probably obscured by the development of age-related degenerative changes unrelated to axSpA. With increasing age, the prevalence of osteoarthritis increases. According to a large register-based study, the prevalence of peripheral joint osteoarthritis in Sweden in 2017 was 11% for males and 17% for females, with 87% of the included patients being ≥50 years old.^29^ Data on axial osteoarthritis in Europe are very scarce, and validated criteria are lacking. A small US study comparing patients with nodal osteoarthritis and PsA showed that the US chronicity score in the lower extremities did not differ between the groups.^30^ Indeed, structural changes at the entheses are not specific to SpA but occur to varying degrees and, in some studies, with high prevalence in healthy controls and patients with metabolic syndrome.^27 28 31^ Furthermore, diffuse idiopathic skeletal hyperostosis (DISH) is a condition characterised by pathological bone formation in the spine and peripheral entheses. The prevalence of DISH increases with advancing age and varies greatly in different studies depending on the methodology.^32^ Simultaneous occurrence of AS and DISH has been reported, and sometimes DISH is misdiagnosed as AS.^33 34^ Radiographs of peripheral entheses in DISH show a high prevalence of bone formation in sites typically affected in SpA,^35^ and one US study reported significantly higher prevalence of enthesophytes in the knees and feet in patients with DISH compared with patients with lower limb osteoarthritis.^36^

We found no significant association between the US damage score and physical activity, or occupational activity among the subgroup still working. In contrast, Bakirci *et al* reported that higher physical activity, assessed by the International Physical Activity Questionnaire (IPAQ), correlated with a higher US damage score in healthy individuals, while Wervers *et al* observed no link between activity avoidance and US damage score in patients with psoriasis.^26 27^ IPAQ provides a broader assessment of activities, including leisure time but also domestic, work-related and transport-related activities, which may partly explain the discrepancies.

We do not have an age-matched control group to compare our results with. Of the four previous studies on US structural entheseal changes and the association with spinal bone formation in SpA, one study included healthy controls. Aydin *et al* found a higher median US enthesophyte score at the Achilles tendon insertion in patients with AS compared with healthy controls overall and in males.^14^ In the present study, we found no difference in the prevalence of structural lesions between males and females with r-axSpA. However, males had a higher US damage score compared with females, and male sex was independently associated with a higher US damage score. Male sex is associated with a higher burden of spinal bone formation, but little has been published previously on sex differences regarding US damage lesions.^37^ Our finding is in line with the studies by Solmaz *et al* on axSpA and Bakirci *et al* on healthy controls, both reporting an independent association between male sex and higher US damage scores.^17 27^ Ruyssen-Witrand * et al* and Polachek *et al* did not investigate sex differences.^15 16^ The underlying reasons why females exhibit a phenotype with less spinal bone formation and potentially less entheseal structural changes remain unclear. In this cohort, sex-related differences that may contribute include a higher BMI and a greater proportion of HLA-B27 positivity in males. The substantially lower mSASSS observed in females, combined with their smaller numbers, likely reduced the ability to detect an association between spinal bone formation and US damage score in this subgroup.

In the present study, 92% of the patients had a structural US lesion, with enthesophytes being the most common lesion. The most common entheseal sites for enthesophytes were at the insertion of the quadriceps tendon (85.5%), followed by Achilles (54%) and the insertion of triceps (34%). Of the previous studies on the association between entheseal structural changes and spinal bone formation, only Ruyssen-Witrand *et al* reported the frequencies of US changes at the different entheseal sites. In their young population, the frequencies of enthesophytes or calcifications were much lower than in our present study, and there were small differences between the examined sites (Achilles 9%, lateral epicondyle 5%, proximal and distal patellar insertion 4% each).^16^ In a recent study of the lower extremities in 224 patients with axSpA, Achilles (60% of patients) and quadriceps (51%) were the entheseal sites with the highest prevalence of enthesophytes/calcifications.^38^ Two smaller studies on axSpA that included the lateral epicondyle also found the insertion of the Achilles or quadriceps tendons as the most prevalent sites for enthesophytes.^39 40^ The same distribution pattern, with the insertions of Achilles and quadriceps tendons being the most common entheseal sites expressing enthesophytes, can be observed in studies on healthy controls and controls with fibromyalgia and osteoarthritis.^27 38 41^ Further, the prevalence of enthesophytes/calcifications among the healthy controls ranged, at the most prevalent site (Achilles), from 41% to 79%,^27 41^ whereas the prevalence among the larger group of controls with fibromyalgia or osteoarthritis was 57%.^38^ The high occurrence of chronic changes in controls and the shared pattern with axSpA patients regarding commonly involved entheseal sites exposed to high amounts of mechanical stress points to a response to mechanical forces that develop over time rather than a disease-specific event.

There are some limitations to our study. The cross-sectional analyses cannot reveal causal effects on the development of structural damage over time. Enthesophytes can vary greatly in size and number at an entheseal site, and a dichotomous score does not capture this. Our smaller sample size, compared with three prior studies on this topic, may have limited the statistical power to detect significant associations, particularly among females, who exhibited considerably lower mSASSS values compared with males. Changes at the plantar fascia were difficult to assess in males, with data from this site missing for several males. The reliability analysis was done on stored images, whereas the scoring was done in real-time. US examinations were performed by a single sonographer; consequently, inter-reader reliability could not be evaluated. Physical activity was assessed using a modified version of the LTPAI (excluding activities with no impact on breathing), a questionnaire validated for fibromyalgia, and we did not capture all domains of activity.^21^ Objective methods were not used, and self-reported physical activity might be biased. Additionally, temporal variations in physical activity were not captured, as participants reported their activity levels only for the past month. Nevertheless, recalling physical activity over a longer duration is challenging and may further compromise data accuracy. By analysing current occupational activity, we did not capture the cumulative mechanical stress from previous employment. We did not examine an age-matched control group for comparison, which limits the interpretation of the results and the ability to differentiate between findings related to axSpA and normal ageing. Patients with nr-axSpA were not included; consequently, findings cannot be generalised to such individuals or younger patients. Osteoarthritis and DISH were not evaluated, both of which are potential confounders that could obscure the relationship between the US damage score and spinal bone formation. The mean interval of 200 days between the US examination and radiography is not likely to have influenced the results, given the typically slow progression rate of mSASSS.

The strength of this study is a well-characterised cohort of patients with the possibility to adjust for important confounders known to affect structural changes at the entheses and spinal bone formation.

## Conclusion

This cross-sectional US study on structural entheseal changes in patients with long-standing r-axSpA showed a univariate association between such changes and spinal bone formation, particularly in males. The associations did not remain overall when the models were adjusted for age or symptom duration. The most probable explanation is that the development of enthesophytes and calcifications at the entheses is partly related to normal ageing and the development of osteoarthritis and that the older age in our cohort obscures the association found in previous studies, mainly conducted on younger patients. Future longitudinal studies are awaited to establish the relationship between the development of US structural changes and spinal new bone formation.

## Supplementary material

10.1136/rmdopen-2025-006388 online supplemental file 1

## Data Availability

Data are available upon reasonable request.
