# Supplementary material for: Relationship between ultrasound damage score of peripheral entheses and spinal bone formation in long-standing radiographic axial spondyloarthritis
Source: RMD Open. 2026 Mar 4;12(1):e006388. doi: 10.1136/rmdopen-2025-006388 (PMC12970138; doi:10.1136/rmdopen-2025-006388)
Supplement: online supplemental file 1 [file rmdopen-12-1-s001.pdf]

Supplementary File. The relationship between ultrasound damage score of peripheral entheses and spinal bone formation in long-standing radiographic axial spondyloarthritis by A. Deminger et al.

Supplementary Table 1.

Multivariable negative binomial regression analyses not adjusted for age, assessing factors associated with ultrasound damage score in 169 patients with radiographic axial spondyloarthritis with spinal radiographs.

	Total group, n = 169<br>RR (95% CI)	*p*-value
Intercept	2.19 (1.29 to 3.73)	**0.004**
Sex, male	1.25 (1.05 to 1.56)	**0.045**
BMI, kg/m^2	1.01 (0.99 to 1.03)	0.32
Log (mSASSS + 1), score	1.27 (1.07 to 1.51)	**0.007**

RR is the rate ratio (Exp (β)), where β models the natural logarithm of the expected count in the ultrasound damage score.

P-values ≤ 0.05 are highlighted in bold.

BMI, body mass index; Log, log-transformed; mSASSS, modified Stoke Ankylosing Spondylitis Spinal Score

Supplementary Table 2. Characteristics and medication in 173 patients with radiographic axial spondyloarthritis by quartiles of age

	Quartile 1 (Q1) n= 44	Quartile 2+3 (Q2+Q3) n = 86	Quartile 4 (Q4) n = 43
**Demographic variables**			
Sex, male	26 (59)	47 (55)	21 (49)
Age, years	38 (36 to 43)	54 (50 to 60)	70 (69 to 73)
BMI, kg/m^2	24.6 (22.7 to 26.9)	27.2 (23.8 to 30.7)	27.5 (24.8 to 29.8)
Current or past smoking, yes	15 (34)	44 (51)	24 (56)
Physical activity, h/week	4.0 (1.1 to 8.0)	4.0 (2.0 to 5.6)	3.0 (2.0 to 5.0)
**Disease-related variables**			
Symptom duration, years	16 (13 to 22)	29 (19 to 36)[a]	43 (36 to 50)[b]
HLA-B27 positive, yes	40 (91)	74 (86)	36 (84)
BASFI, score	1.2 (0.6 to 2.7)	2.3 (1.1 to 4.4)	3.8 (2.2 to 5.2)
BASDAI, score	2.6 (1.6 to 3.6)	3.5 (1.8 to 5.5)	3.6 (2.0 to 5.4)[b]
ASDAS, score	1.9 (1.2 to 2.4)	2.0 (1.3 to 2.9)	2.4 (1.6 to 2.7)[b]
CRP, mg/L	2.0 (0.5 to 5.8)	3.0 (1.0 to 5.3)	3.0 (2.0 to 8.0)
mSASSS, (0-72)	2.0 (0 to 6.0)[c]	9.0 (0 to 24.0)[d]	20.5 (5.8 to 51)[b]
≥ 1 syndesmophyte, yes	12 (29)[c]	45 (54)[d]	30 (71)[b]
Use of NSAIDs, yes	29 (66)	58 (67)	28 (65)
Use of TNFi, yes	11 (25)	23 (27)	4 (9)
Total US damage score	2.0 (0 to 4.0)	3.0 (2.0 to 5.0)	6.0 (4.0 to 7.3)

Values are presented as median (25th to 75th percentile) or number (%)

[a]n = 85, [b]n = 42, [c]n = 43, [d]n = 84

ASDAS, Ankylosing Spondylitis Disease Activity Score; BASDAI, Bath Ankylosing Spondylitis Disease Activity Index; BASFI, Bath Ankylosing Spondylitis Functional Index; BMI, body mass index; CRP, C-reactive protein; HLA-B27, human leukocyte antigen B27; mSASSS, modified Stoke Ankylosing Spondylitis Spinal Score; NSAID, non-steroidal anti-inflammatory drug; TNFi, tumor necrosis factor inhibitor; US, ultrasound

Supplementary Table 3. Univariate negative binomial regression analyses assessing factors associated with ultrasound damage score in 169 patients with radiographic axial spondyloarthritis and spinal radiographs by quartiles of age

Age min – max, years	Quartile 1 (Q1)<br>27 – 45<br>RR (95% CI)	*p*-value	Quartile 2+3 (Q2+Q3)<br>46 - 66<br>RR (95% CI)	*p*-value	Quartile 4 (Q4)<br>67 – 81<br>RR (95% CI)	*p*-value
Sex, males	2.05 (1.11 to 3.79)	**0.021**	1.54 (1.18 to 2.01)	**0.001**	1.28 (1.00 to 1.65)	0.050
Age, years	0.98 (0.91 to 1.06)	0.63	1.01 (0.99 to 1.03)	0.33	1.01 (0.97 to 1.05)	0.65
BMI, kg/m^2	0.99 (0.92 to 1.08)	0.87	1.02 (1.00 to 1.05)	0.056	1.00 (0.98 to 1.03)	0.93
Current/past smoking, yes	1.03 (0.54 to 1.97)	0.93	0.97 (0.74 to 1.27)	0.83	0.93 (0.72 to 1.21)	0.60
Log Physical activity+1, h/week	0.74 (0.33 to 1.67)	0.47	0.85 (0.54 to 1.34)	0.48	0.97 (0.61 to 1.56)	0.91
Symptom duration, years	1.01 (0.96 to 1.08)	0.63	1.00 (0.99 to 1.02)	0.81	0.99 (0.98 to 1.00)	0.060
HLA-B27 positive, yes	3.45 (0.87 to 13.78)	0.079	0.80 (0.55 to 1.15)	0.22	0.99 (0.70 to 1.39)	0.94
Use of TNFi, yes	1.61 (0.83 to 3.11)	0.16	0.92 (0.68 to 1.25)	0.61	1.20 (0.80 to 1.81)	0.37
BASDAI, score	0.92 (0.78 to 1.10)	0.37	1.01 (0.95 to 1.07)	0.87	0.97 (0.90 to 1.05)	0.47
CRP, mg/L	0.94 (0.87 to 1.01)	0.11	1.01 (0.99 to 1.04)	0.35	0.99 (0.96 to 1.01)	0.22
Log mSASSS+1, score	1.11 (0.56 to 2.20)	0.77	1.35 (1.10 to 1.64)	**0.004**	1.02 (0.82 to 1.25)	0.89

RR is the rate ratio (Exp (β)), where β models the natural logarithm of the expected count in the ultrasound damage score.

P-values ≤ 0.05 are highlighted in bold

BASDAI, Bath Ankylosing Spondylitis Disease Activity Index; BMI, body mass index; CRP, C-reactive protein; HLA-B27, human leukocyte antigen B27; Log, log-transformed; mSASSS, modified Stoke Ankylosing Spondylitis Spinal Score; TNFi, tumor necrosis factor inhibitor

Supplementary Table 4. Multivariable negative binomial regression analysis assessing factors associated with ultrasound damage score in patients with radiographic axial spondyloarthritis and spinal radiographs in age quartiles 2 and 3 combined.

	Quartile 2 + 3 (Q2+Q3)<br>n = 84<br>RR (95% CI)	*p*-value
Intercept	2.04 (1.08 to 3.83)	**0.028**
Sex, male	1.38 (1.04 to 1.83)	**0.027**
BMI, kg/m^2	1.01 (0.99 to 1.04)	0.29
Log mSASSS+1, score	1.18 (0.95 to 1.47)	0.15

RR is the rate ratio (Exp (β)), where β models the natural logarithm of the expected count in the ultrasound damage score.

P-values ≤ 0.05 are highlighted in bold

BMI, body mass index; Log, log-transformed; mSASSS, modified Stoke Ankylosing Spondylitis Spinal Score

Supplementary Table 5. Sensitivity analyses using the ultrasound damage score excluding the plantar fascia as the dependent variable in multivariable negative binomial regression analyses in the total group and males.

	Total group,<br>n= 169<br>RR (95% CI)	*p*-value	Males,<br>n = 92<br>RR (95% CI)	*p*-value
Intercept	0.64 (0.33 to 1.21)	0.17	1.53 (0.80 to 2.94)	0.20
Sex, male	1.51 (1.23 to 1.82)	**<0.001**	NA	
Age, years	1.02 (1.02 to 1.03)	**<0.001**	1.02 (1.01 to 1.03)	**0.003**
BMI, kg/m^2	1.01 (0.99 to 1.03)	0.34	NM	
Log (Physical activity + 1), h/week	NM		0.86 (0.58 to 1.26)	0.44
BASDAI, score	NM		1.04 (0.97 to 1.11)	0.28
Log (mSASSS + 1), score	1.01 (0.85 to 1.20)	0.95	1.08 (0.85 to 1.37)	0.52

RR is the rate ratio (Exp (β)), where β models the natural logarithm of the expected count in the ultrasound damage score.

P-values ≤ 0.05 are highlighted in bold.

BASDAI, Bath Ankylosing Spondylitis Disease Activity Index; BMI, body mass index; h, hours; Log, log-transformed; mSASSS, modified Stoke Ankylosing Spondylitis Spinal Score; NA, not applicable; NM, not used in the model due to *p*-value > 0.1 in the univariate analysis.
